# A critical analysis of the current state of virus taxonomy

**DOI:** 10.3389/fmicb.2023.1240993

**Published:** 2023-08-03

**Authors:** Gustavo Caetano-Anollés, Jean-Michel Claverie, Arshan Nasir

**Affiliations:** ^1^Evolutionary Bioinformatics Laboratory, Department of Crop Sciences and C.R. Woese Institute for Genomic Biology, University of Illinois at Urbana-Champaign, Urbana, IL, United States; ^2^Structural and Genomic Information Laboratory (UMR7256), Mediterranean Institute of Microbiology (FR3479), IM2B, IOM, Aix Marseille University, CNRS, Marseille, France; ^3^Moderna, Inc., Cambridge, MA, United States

**Keywords:** classification, evolution, holobiont, horizontal genetic transfer, ICTV, reticulation, virus origin

## Abstract

Taxonomical classification has preceded evolutionary understanding. For that reason, taxonomy has become a battleground fueled by knowledge gaps, technical limitations, and *a priorism*. Here we assess the current state of the challenging field, focusing on fallacies that are common in viral classification. We emphasize that viruses are crucial contributors to the genomic and functional makeup of holobionts, organismal communities that behave as units of biological organization. Consequently, viruses cannot be considered taxonomic units because they challenge crucial concepts of organismality and individuality. Instead, they should be considered processes that integrate virions and their hosts into life cycles. Viruses harbor phylogenetic signatures of genetic transfer that compromise monophyly and the validity of deep taxonomic ranks. A focus on building phylogenetic networks using alignment-free methodologies and molecular structure can help mitigate the impasse, at least in part. Finally, structural phylogenomic analysis challenges the polyphyletic scenario of multiple viral origins adopted by virus taxonomy, defeating a polyphyletic origin and supporting instead an ancient cellular origin of viruses. We therefore, prompt abandoning deep ranks and urgently reevaluating the validity of taxonomic units and principles of virus classification.

## Introduction

**Figure 1:**
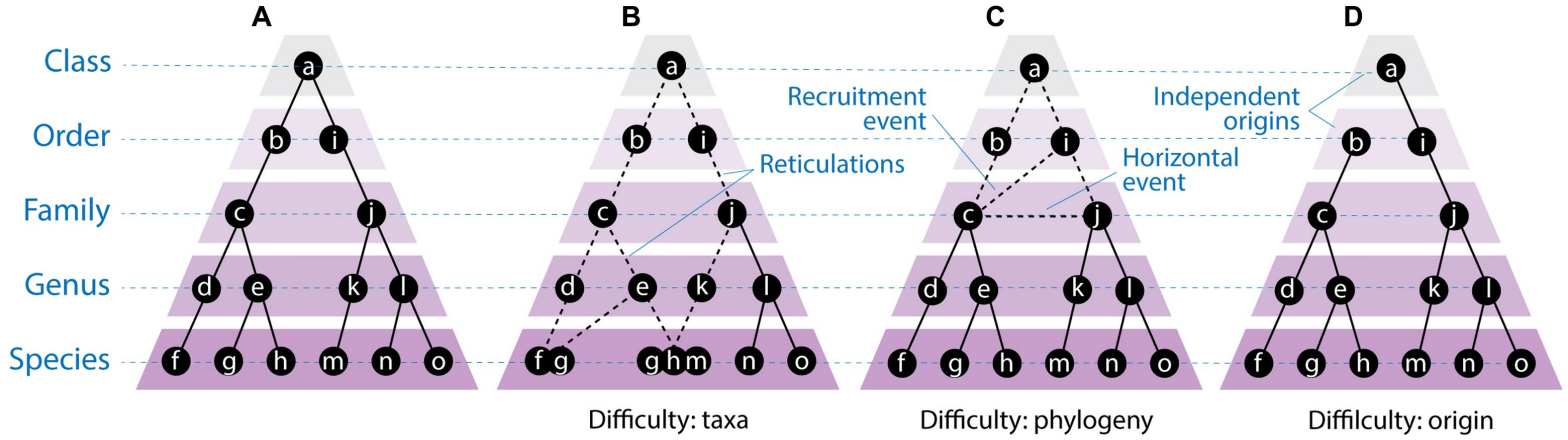
Matching taxonomies to evolution. The endeavor **(A)** may prove difficult in the presence of taxonomic terminal units that are holobionts **(B)**, phylogenies with reticulations (dashed lines) caused by horizontal gene transfer (line connecting taxa c and j) or recruitment (line connecting taxa c and i) **(C)**, or the existence of independent origins that break up monophyletic relationships **(D)**. Note that reticulations at higher rank levels enhance the chances of multiple origins in evolution.

In biology, taxonomy is the science of naming, describing and classifying biological entities. Since its formal inception with Carolus Linnaeus almost 300 years ago, the initial ranked system of organismal categorization has progressed based on the premise that there is a ‘natural’ evolutionary relationship established between the organisms that are being classified. Currently, the accepted taxonomy approach incorporates phylogenetic relationships as crucial factor in the proposal of taxonomic groups, and, in absence of evolutionary information or presence of confounding evidence, the field employs a variety of other characteristics (often phenotypic in nature) to assist in the taxonomic endeavor (Godfray, 2002; Padial et al., 2010; Hugenholtz et al., 2021). However, taxonomic classification has been a battleground, mainly because classification has preceded our understanding of both the evolutionary relationships that exist between organisms and the evolutionary drivers of those relationships. Here, battleground is used as metaphor of different, often dissenting, opinions shaping belief and politics of scientific discourse (Bryson, 2003) that continue to unfold in the post-genomic era fueled by knowledge gaps, technical limitations, and the shortcomings of *a priorism*, i.e., epistemic justifications that are independent from experience. The following three examples illustrate battleground challenges that lay ahead (Figure 1).

### Taxonomic units

Species have been considered the units of both taxonomic classification and phylogenetic reconstruction because they originate from processes of population variation and reproductive isolation that ultimately resolve into the splitting branches of the Tree of Life (ToL) (Hey et al., 2005). Yet, these taxonomic units (taxa) remain controversial and not well defined. To begin with, most organisms are ‘holobionts’ (Meyer-Abich, 1943; Jefferson, 1994; Zilber-Rosenberg and Rosenberg, 2008; Rosenberg and Zilber-Rosenberg, 2013), organismal communities organized around individual hosts that behave as units of biological organization (Bordenstein and Theis, 2015; Theis et al., 2016). These communities exhibit synergistic phenotypes that impact their anatomy, physiology, reproduction, and behavior and impinge on their fitness, pushing interacting organisms to evolve in coordination. In fact, the ‘hologenomes’ of these communities represent comprehensive and integrated gene systems, challenging the concepts of ‘individuality’ (Gilbert et al., 2012) and ‘organismality’ (Queller and Strassmann, 2009). Hologenomes integrate all mechanisms of mutation across many genomes, inducing inter-genome covariation and epistasis. For example, complex multicellular organisms such as humans depend on their microbiomes for their well-being but their genomic makeup is in constant flux, subject to horizontal gene transfer events occurring at different temporal scales and mediating a ‘genetic crosstalk’ that moves genes throughout the human body (Jeong et al., 2019). Similarly, coral reefs are home to a wide diversity of marine invertebrates engaging in tight symbiotic interactions with dinoflagellate, bacterial and viral communities (Webster and Reusch, 2017). Coral holobionts support a quarter of all described marine species, mostly driven by the presence of photosymbionts (Bourne et al., 2013; Webster and Reusch, 2017). Remarkably, changes in the relative abundance of organisms in the coral communities are analogous to the effects of host gene duplication, shuffling and exchange, facilitating coral’s metabolic capacity through metabolic handoffs and genetic exchange (van Oppen and Medina, 2020). These two examples suggest that species are not autonomous entities that evolve in isolation. Instead, they represent tightly-knit collectives spanning organisms from all major domains of life plus viruses. Since holobionts are recognized as dynamic and interconnected systems, exchange of genetic material, metabolites, and signals occurring within different tissues and organs of the host, will blur the line between the host and its symbiotic partners. This makes determining the exact ‘boundaries’ of a holobiont difficult. Boundary ambiguities in holobionts challenge the study of component contributions and interactions, raising questions of evolutionary and ecological significance. For example, various holobionts can exhibit distinct co-evolutionary histories, with some being more recently or anciently formed, each exerting varying degrees of influence on the evolving collectives. We note, however, that the ‘holobiont concept’ may be context dependent. It may make more sense to treat organisms as holobionts for both ecological and evolutionary perspectives, but not so for medicine, where the objective may be to design medicines and vaccines for the host rather than its collectives. We are therefore confronting both a knowledge gap and a conceptual framework that requires taxa be considered units of both evolution and biological organization. This undermines the feasibility of using species as taxonomic units.

### Phylogenies

The recognition of the wide-ranging evolutionary impact of horizontal gene transfer over two decades ago (Doolittle, 1999) challenged the use of phylogenetic trees as evolutionary ground plans (phylogenies) and demanded the reconstruction of phylogenetic networks that would account for the existence of reticulations (net-like evolutionary patterns) caused by events of lateral transfer, hybridization, recombination, reassortment, fusion, and endosymbiosis (Mindell and Meyer, 2001), as well as other entanglements (e.g., recruitment) that are ubiquitous in biology (Caetano-Anollés et al., 2022). Standard evolutionary ontologies of nested hierarchies are now compromised by the fact that their dynamics is not driven solely by vertical descent, requiring instead a new more pluralistic ‘processual’ ontology that is network based (Bapteste and Dupré, 2013). Formalizing evolving network views is also challenging at more technical bioinformatic and computational levels. Despite advances in high-throughput computation, reconstruction of phylogenetic networks from sequence and phenotypic data remains an intimidating task (Huson et al., 2010; Morrison, 2011). Three general types of phylogenetic methods have been implemented. One type generates networks with distance matrices that summarize conflicting phylogenetic information. These methods include the popular Neighbor-Net (Bryant and Moulton, 2004) and Split-Decomposition (Bandelt and Dress, 1992) approaches. They are fast but can be inaccurate and do not build phylogenetic histories. A second type reconstructs networks from weighted triplets, quartets and sextets, all of which harbor more phylogenetic information than distances. These methods make use of parsimony and local maximum likelihood implementations. An example is the parsimony-based QS-net (Tan et al., 2019), which extends the popular Quartet-Net (Yang et al., 2013) to sets of six taxa. A third type reconstructs networks directly from character data using search methods and optimality criteria. These more traditional phylogenetic approaches are often helped by optimizing both trees and networks. Examples include the reconstruction of soft-wired networks with maximum parsimony (Wheeler, 2015), maximum pseudo-likelihood under incomplete lineage sorting (implemented in PhyloNetworks; Solís-Lemus et al., 2017), and deep coalescence minimization from multilocus data (implemented in PhyloNet; Wen et al., 2018). These methods are computationally inefficient and often overestimate reticulations. In general, reconstruction performance decreases with increasing reticulation levels and network reconstruction becomes increasingly more difficult with increasing number of taxa. The inability to accommodate the expected large number of reticulations at global levels, especially those embodying deep branches and multiple origins, compromises the technical feasibility of using phylogenetic relationships to support taxonomic classifications. This challenges the entire taxonomic and phylogenetic enterprise.

### Origins

The problem of building a rooted ToL is of great significance for the validity of integrating taxonomic relationships and for the definition of deep ranks. Rooting a canonical ToL implies identifying and pulling down the branch that holds the last universal common ancestor (LUCA), which imposes an arrow of time on the phylogeny. Despite its significance, the ToL research field has been plagued by the shortcomings of *a priorism* in the form of *ad hoc* and auxiliary assumptions, especially those that are concerningly ‘argumentative’ (Caetano-Anollés et al., 2018). This hampers understanding of deep evolutionary relationships that unify organismal groups (Gouy et al., 2015; Kapli et al., 2020). In addition, building a ToL that is truly representative of the entire biodiversity of our planet is challenged by the enormous scope of the endeavor and the limits of phylogenetic analyses. While about 2 million species of cellular organisms have been named (e.g., Mora et al., 2011), conservative estimates consider there may be more than a trillion species on Earth (Locey and Lennon, 2016; Louca et al., 2019), not to mention the unknown ‘dark matter’ representing organisms that have not been surveyed or cannot be cultivated (only recently added to ToL reconstructions; Hug et al., 2016). For example, a community effort to integrate thousands of phylogenies describing the evolution of about 2.3 million taxa reveal patchiness, gaps of knowledge, and important conflicts (Hinchliff et al., 2015). The evolutionary origins of a number of highly sampled and diverse organismal groups remain contested, including fungi, microbial eukaryotes, bacteria and archaea. In particular, the early diverging animal and eukaryotic groups retain multiple conflicting resolutions. For example, the basal placement of either *Porifera* (sponges; Redmond and McLysaght, 2021) or *Ctenophora* (comb jellies; Whelan et al., 2017; Schultz et al., 2023) in trees of metazoan species remains contested. Defining microbial taxa continues to be problematic in these studies because of rampant horizontal gene exchange and lack of clarity on what is a microbial species. The monophyletic relationship of Archaea remains contentious, as well as its links to Eukarya. Even the depiction of the ToL as a three-domain system heralded by the school of Carl R. Woese (Woese et al., 1990) has been contested, likely fueled by technical and conceptual difficulties related to the use of standard alignment-dependent sequence methodologies of phylogenetic reconstruction (Nasir et al., 2021a). All of these limitations have in particular complicated prokaryotic taxonomy and nomenclature (Hugenholtz et al., 2021). Finally, because ‘outgroups’ cannot be used to root the ToL or ‘groups of interest’ (ingroup taxa) that have non-existent, unknown or distant outgroups, other approaches must be used to dissect the origins of cellular complexity (Caetano-Anollés et al., 2018). In this context, rooting alignment-free phylogenies with Weston’s rule (Weston, 1988) appears a promising approach. Examples include the evolutionary analysis of structural domains (Caetano-Anollés et al., 2021) or homologies in paralogous single-nucleotide polymorphisms (SNPs) of whole-genome sequences (Pearson et al., 2013). Importantly, these approaches are “alignment-free” and thus inherently protect from many of the biases that may result from alignment-dependent methods (e.g., how to treat gaps, presence of fast-evolving taxa, co-dependency of sequence sites to form a structure). Unfortunately, the strategy has been underutilized.

While these selected three battleground problems (taxa, phylogeny and origins) illustrate the difficulties of building taxonomies from evolutionary information, there are more serious limitations that hamper the endeavor. One of them is the exclusion from the ToL of a group of biological entities of planetary significance, the viruses. A ToL is not a ToL if it excludes a major taxonomic group. While there is still much to debate about the validity of including viruses in the ToL (Harris and Hill, 2021), the fact that this tree is not a ‘universal’ phylogeny (a uToL) stands as a central conceptual problem for biology. It also stands as a conceptual problem for virus taxonomy, which appears divorced from a taxonomy of the cellular world. Building on previous elaborations (Claverie, 2020), here we dissect the feasibility of constructing an accurate taxonomy of viruses that mimics their ancient origins and evolution.

## A taxonomy of viruses and the problem of deep taxonomic ranks

**Figure 2:**
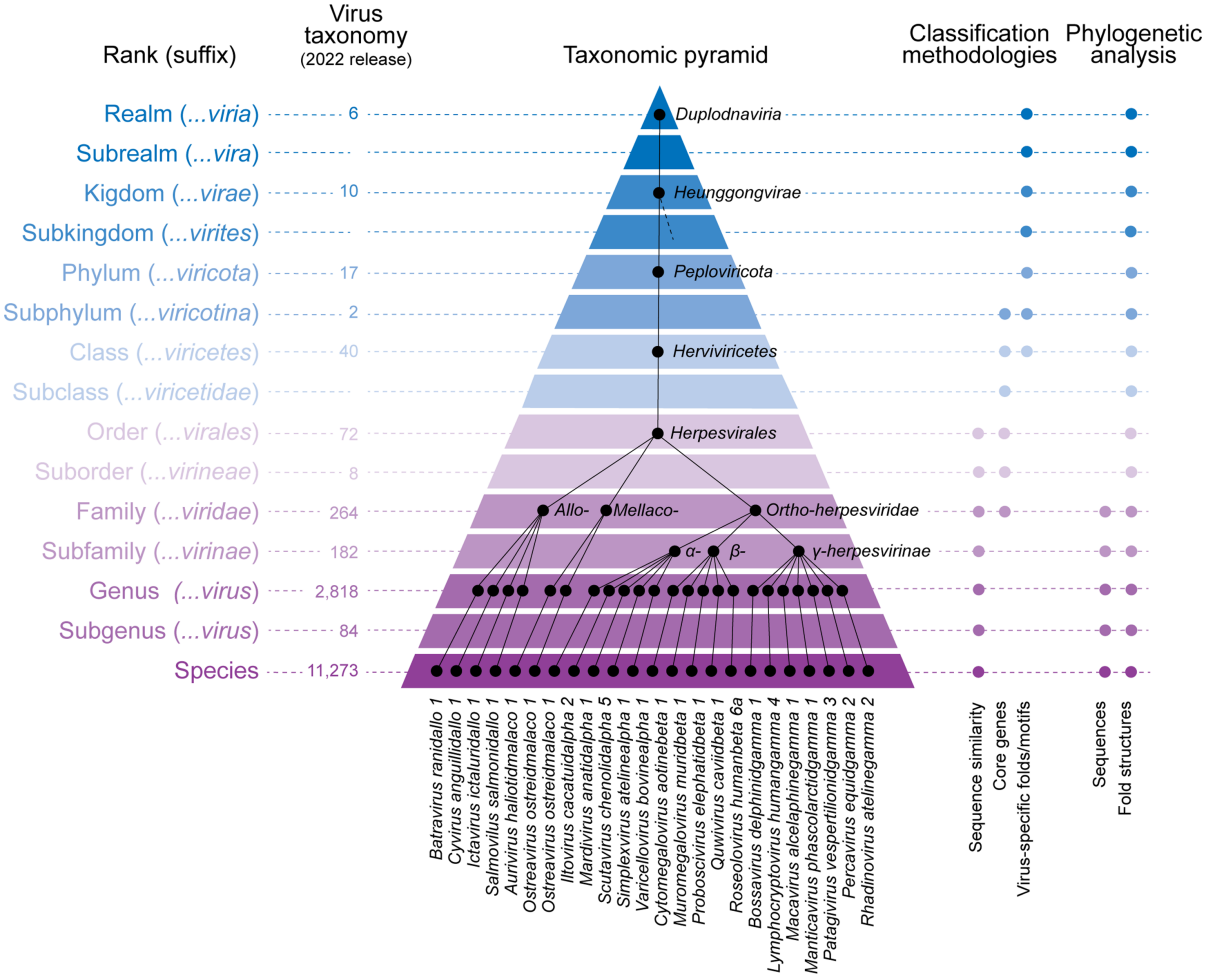
The current virus taxonomy is a 15-ranked system that can be visualized as a taxonomic pyramid when phylogenetic relationships are mapped onto the ranked classification system. The example pyramid shows a classification of the phylum ‘Peploviricota’, which hosts the herpesviruses. Note that only one species per genus illustrates the 133 that currently map to the different genera.

Initial efforts to produce an all-encompassing virus taxonomy began in the 1960s with a formal systematic classification scheme that grouped viruses into taxonomic categories based on shared viral characteristics (Lwoff et al., 1962), including virion morphology (Wildy, 1961), nucleic acid genetic material (Cooper, 1961), and physical attributes such as sensitivity to low pH and virus shape and symmetry (Hamparian et al., 1963). The first accepted taxonomic system grouped viruses into one phylum (‘vira’) with two subphyla containing RNA viruses (‘ribovira’) or DNA viruses (‘deoxyvira’), followed by classes defined by virion symmetry. These classes were further subdivided into orders, families, genera and species (types), lower ranks that are still in use today. An *International Committee on Nomenclature of Viruses* (ICVN) established in 1966, and renamed *International Committee on Taxonomy of Viruse*s (ICTV) in 1974, released the first ratified virus taxonomy in 1971 (MSL #1). It had 2 families, 43 genera, and 290 type members (species). Release 1990 (MSL #11) included an order (plus 40 families, 9 subfamilies, 137 genera, and 1,290 species), release 2018 (MSL #34) included a realm, a phylum and 2 subphyla (plus 6 classes, 14 orders, 150 families, 79 subfamilies, 1,019 genera, and 5,560 species), and release 2019 (MSL #35) included 4 realms and 9 kingdoms (plus 16 phyla, 2 subphyla, 36 classes, 55 orders, 8 suborders, 168 families, 103 subfamilies, 1,421 genera, 68 subgenera, and 6,590 species). The current ICTV taxonomy (release 2022, MSL #38) now adopts an expanded 15-ranked classification system (International Committee on Taxonomy of Viruses Executive Committee, 2020) with 6 realms and 10 kingdoms hosting 11,273 viral species (Figure 2). We note the rapid higher rank complexification of the virus taxonomy in the course of a few years triggered by the construction of a phylogeny of RNA viruses from an alignment of RNA-directed RNA polymerases (RdRP; Wolf et al., 2018), and the adoption of one out of several hypotheses of viral origins (Koonin et al., 2020) despite significant evidence supporting countering hypotheses (Nasir and Caetano-Anollés, 2015; Mughal et al., 2020). The new ranks brought with them new (sometimes unpronounceable) names (e.g., *Heunggongvirae*, *Chunqiuviricetes*, *Huolimaviricetes*, *Pokkesviricetes*, *Stelpaviricetes*) that obscure any reference to pioneering scientists or virological history preceding this naming frenzy. For example, the introduction of Mimivirus-related viruses cite proponents of the *Megaviricetes* and *Imitervirales* taxonomic ranks, none of which ever isolated a virus. The higher ranks brought with them intriguing cases, such as those of the *Polyomaviridae* and *Papillomaviridae* that are now classified within the *Monodnaviria* (hence ssDNA viruses) while their genomes are dsDNA. This will surely confuse newcomers to the field of virology.

**Figure 3:**
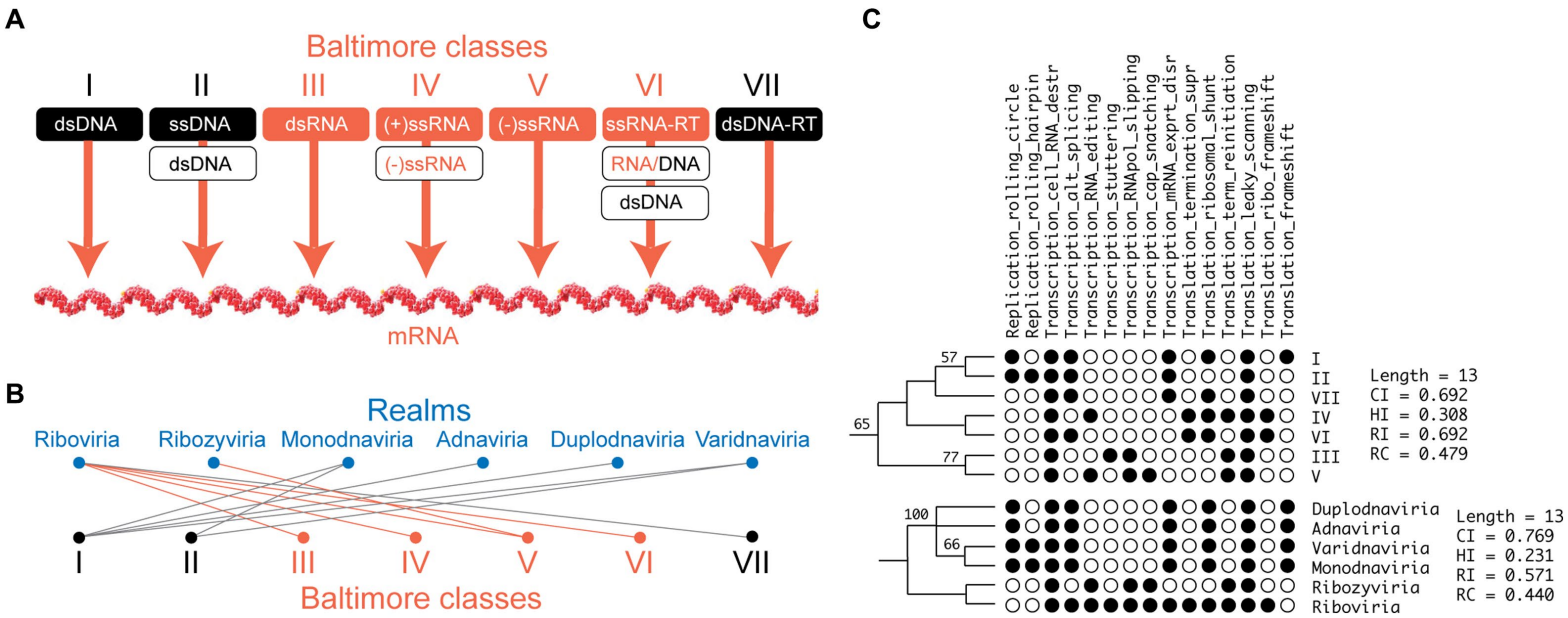
Comparing the Baltimore classification of viruses and the ranking of realms uncovers evolutionarily entangled systems. **(A)** The seven Baltimore classes describe processes of information transfer that lead to mRNA molecules necessary for translation into viral proteins. **(B)** A bimodal network mapping realms to Baltimore classes shows the entangled relationships between the two classification schemes. **(C)** A phylogenetic reconstruction of a tree of Baltimore classes and a tree of realms from viral traits related to replication, transcription and translation reveals comparable evolutionary histories. CI, consistency index; HI, homoplasy index; RI, Retention index; RC, Rescaled consistency index.

The introduction by the ICTV of the ‘realm’ concept in 2018 changes the entire virus taxonomy landscape. It tries to equate this evolutionarily deep viral grouping to the concept of ‘domain’ in cellular organismal classification. It also replaces a widely-used but informal albeit clever and scientifically sound classification system introduced by Baltimore (1971) that clustered viruses into seven groups (‘Baltimore classes’) according to the type of nucleic acids present in their genomes and routes of genetic information transfer leading to mRNA and the encoded proteins (Figure 3A). These Baltimore classes represent only a subset of the 35 classes of information transfer (grouped into 17 superclasses and 6 types) that are possible in viruses (Agol, 1974), only 14 of which have materialized in evolution (a fact demanding explanation). While there was an implicit assumption that Baltimore classes represented monophyletic groups of taxa (only recently formalized as a proposal; Gorbalenya, 2018), the recent ICTV overhaul (International Committee on Taxonomy of Viruses Executive Committee, 2020) replaced the 7 Baltimore classes by six realms, which mapped to the Baltimore classes in entangled manner (Figure 3B). This overhaul assumed realms were monophyletic groups based on a small set of virus hallmark genes involved in virus replication (such as RdRPs of *Riboviria*) or virion formation (such as double jelly roll capsid proteins of *Varidnaviria*), when in fact there are no genes that can unify all viruses and significant structural phylogenomic evidence point to their very ancient paraphyletic origin (Nasir and Caetano-Anollés, 2015; Mughal et al., 2020). Since taxonomy is based on a pyramidal structure (Figure 2), there is insistence that realms must represent monophyletic groups (Simmonds et al., 2023). However, monophyly cannot be tested without suitable outgroups, even when using the sequence of proper hallmark genes, and there are no appropriate outgroups for realms (they stand alone as a separate evolutionary groups). Consequently, the assumption that realms represent *bona fide* monophyletic groups awaits confirmation. Without a suitable test, the overhaul also assumed that the realm classification was superior to the Baltimore classification (the null hypothesis) in its ability to portray basal evolutionary relationships. We contend this is not so. A simple phylogenetic reconstruction exercise described in Figure 3C compared the most-parsimonious trees of Baltimore classes and realms reconstructed using 15 phylogenetic characters describing central replication, transcription and translation characteristics that were drawn from annotations by Rampersand and Tennant (2018). Phylogenies rooted using Weston’s rule with the Lundberg criterion showed significant vertical phylogenetic signatures unifying the 7 Baltimore classes or the 6 realms. However, phylogenies also showed realms offered no significant improvement in their ability to decrease tree length (a direct measure of phylogenetic optimality) or homoplasy (an indirect measure of reticulation) measured with the homoplasy index (HI). In fact, while the phylogeny of Baltimore classes was fully resolved and showed marginal-to-moderate support for basal splits, *Adnaviria* and *Duplodnaviria* could not be dissected and basal branching relationships were unsupported in the tree of realms. In the absence of significant phylogenetic improvement and the presence of significant evidence supporting the paraphyletic relationship of viruses, a rationale for complicating virus taxonomy already demands an urgent re-examination.

Currently, no general methodology for virus classification has been officially adopted by the ICTV. Alignment-dependent phylogenetic methods involving nucleic acid and protein sequences support statements of relationships at lower-level ranks (Figure 2), but the techniques have been increasingly used at higher ranks (e.g., Wolf et al., 2018) despite concerning limitations (Holmes and Duchêne, 2019). Conversely, sequence similarities, core genes that are most often present, or virus-specific fold/motifs have been used to define higher taxonomic ranks (Figure 2). While structural phylogenomic analysis of entire virus genomic complements of fold structures can support deep statements of evolutionary relationship at higher rank levels, including phylogenies of all virus groups (Nasir and Caetano-Anollés, 2015; Mughal et al., 2020), these methodologies have not been considered by ICTV. Instead, pairwise sequence comparisons of complete viral genomes have been recommended, especially to demarcate similarity cut-offs for taxa. For higher ranks, alignment-dependent analysis involves single or subsets of about 7–20 hallmark genes holding divergent evolutionary histories constrained by widely divergent fold structures. This makes the threshold approach sequence-level dependent, noisy and dubious. Note that the deeper the taxonomic rank, the smaller the number of homologous genes from which to build phylogenies with sequence alignment-dependent methods. In the case of eukaryotic dsDNA viruses, their number is small, with only 3 present within the phylum *Nucleocytoviricota*, and none within kingdom *Bamfordvirae* (Guglielmini et al., 2019). These ‘core’ genes sometimes exhibit better similarity to homologs from cellular organisms, the inclusion of which makes phylogenies of virus homologs inconsistent. Similarity searching programs such as BLAST or HMMR, measure ‘excess similarity’ in sequence comparisons, a statistical descriptor that approximates homology (Pearson, 2013). However, homology must be confirmed by building multiple sequence alignments, removing unreliable parts of the alignment (filtering), identifying homologous characters, and mitigating uncertainty in homology inference during phylogenetic reconstruction. In the highly reduced genomes of viruses, there are many cases of false homologies that lead to non-sensical phylogenetic inferences when homologies are not adequately sorted. Multiple sequence alignments at borderline significance level may lead to false homolog identification, claiming for example the existence of capsid proteins when these are absent (see Supplementary Figure S2 in Krupovic et al., 2020). In our experience (tested for *Nucleocytoviricota*), the retainment of cellular homologs in BLAST searches provides an objective criterion to delimit a viral family (e.g., using the DNA polymerase gene), reflecting the deep connection of viruses with the cellular world. For viruses, the main limitation is the very small number of recognizable common ‘core’ genes dispersed among highly diverse gene contents that would justify their use in virus classification. For example, the placement of pandoraviruses (>2,500 protein-coding genes) with coccolithoviruses (members of *Phycodnaviridae* with ~500 protein-coding genes) on the same clade (Yutin and Koonin, 2013) based on only 6 ‘cherry picked’ core genes is difficult to justify in the presence of hundreds of other genes, most of which are ORFans and many of which have close cellular homologs. In particular, filtering has been a problematic step in phylogenetic sequence analysis (Tan et al., 2015). Deeper phylogenetic relationships entail more divergent sequences and therefore a need to incorporate an increasing number of gaps in sequence alignment. However, there is no reliable way to treat gaps. State-of-the-art programs such as RAxML (Kozlov et al., 2019) and IQTREE (Minh et al., 2020) treat gaps as missing data or as sites that hold no information (as if they were sequencing errors), a situation that can make likelihood inferences inconsistent (Warnow, 2012). An alternative is to code gaps as an additional character state, e.g., 5th state besides A, G, C, and T in DNA alignments or 21st state besides the 20 amino acids in protein alignments (Dwivedi and Gadagkar, 2009). Unfortunately, while this approach may improve tree reconstruction accuracy, consecutive gaps do not represent characters evolving independently of the other. Instead, evolutionary interactions violate character independence in likelihood-models (Caetano-Anollés et al., 2018) and overweigh characters biasing phylogenetic reconstruction (Chippindale and Wiens, 1994). Even conserved sites violate character independence when they interact with other sites to form folded molecular structures (Nasrallah et al., 2011). Without reliable structural alignment-based benchmarking systems (Iantorno et al., 2014) the uncertainties appear unconquerable. All of these limitations are even complicated by the fact that distinct groups of viruses evolve at different rates depending on gene and genome type, proofreading mechanisms, and genome rearrangements as well as horizontal transfer propensities. For example, the latest atlas of adaptive evolution in different endemic viruses assembled by Kistler and Bedford (2023) shows clear differences in the rates of adaptive evolution in viruses from within the same family (e.g., OC43 and NL63 from Coronaviruses, H3N2 and Influenza B lineages, and Norovirus GII.4).

## Limitations of virus taxonomy

**Figure 4:**
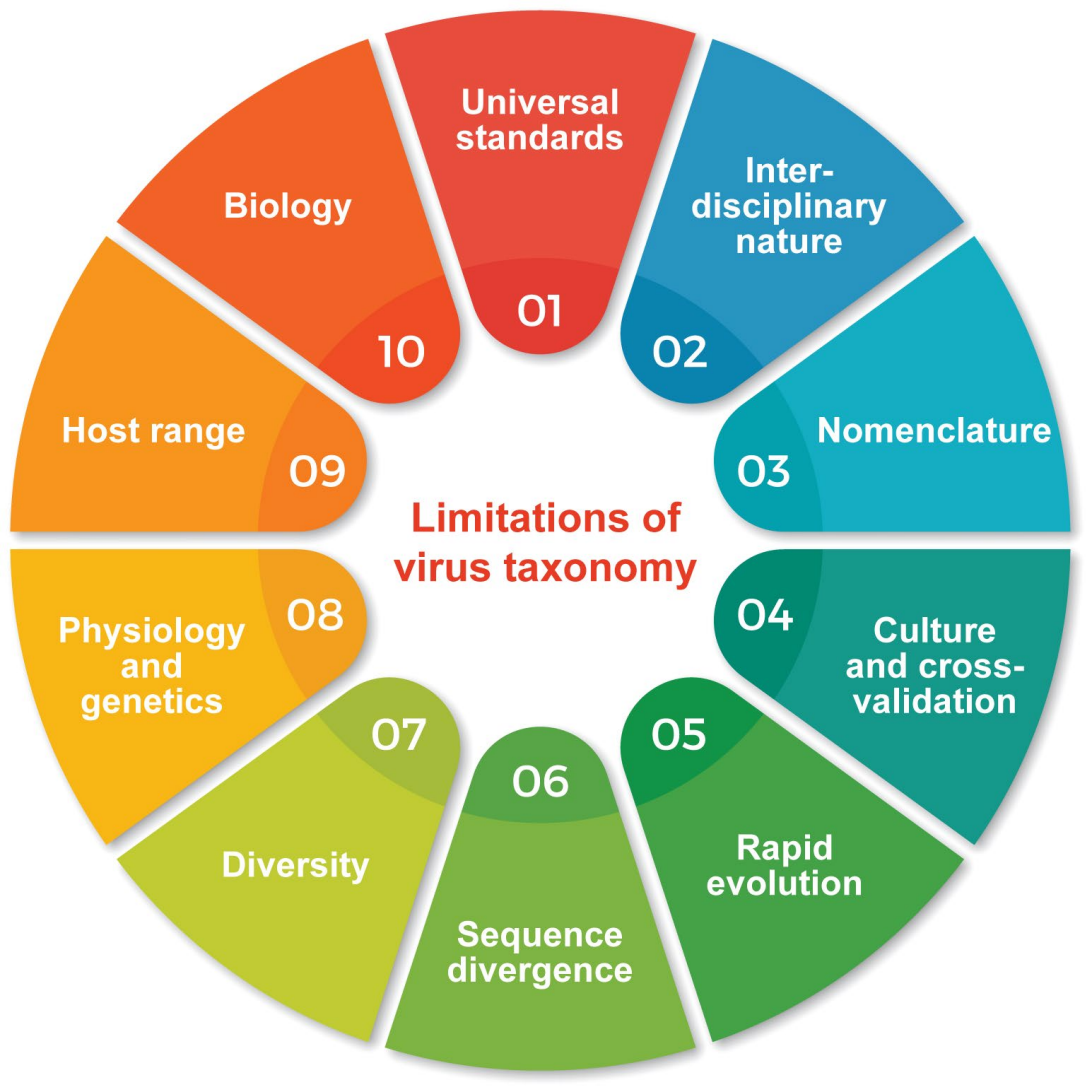
Well-known limitations makes building a virus taxonomy a challenging proposition.

A number of well-known difficulties (Figure 4) makes building a virus taxonomy with classical approaches of classification an already challenging proposition:

1. *Universal standards:* There are no universally accepted standards for virus taxonomy, which can lead to confusion and inconsistency. Unlike other living organisms, viruses do not fit neatly into the traditional classification system, which is based on evolutionary relationships and shared phenotypic characteristics. While most genomes of viruses in ICTV taxonomy have been sequenced and there is acceptance that monophyletic evolutionary relationship should drive classification (Simmonds et al., 2023), viruses are generally classified at ranks other than species and genus level based on a combination of their genetic material, morphology, host range, and other polythetic characteristics, making reconciliation with phylogenetic information difficult across different viral groups (e.g., different viruses that cause hepatitis with different genetic material are often commonly referred to as the Hepatitis viruses and their distinct evolutionary histories are not obvious to the common public). There is also a lack of a clear consensus on the criteria for classification, such as the level of similarity required to define a viral species or the use of phenotypic traits that hold useful phylogenetic information. Furthermore, different informal taxonomic systems are used by different scientific communities, further complicating efforts to establish a universal taxonomy. The absence of standardized virus taxonomy has practical implications for medical research and public health. In the case of the ongoing COVID-19 pandemic, for example, it has been suggested that Omicron be labeled SARS-CoV-3 due to its higher antigenic evolution and immune escape relative to pre-Omicron viruses (Vogel, 2022). This uncertainty can affect efforts tracking the spread of the virus, developing effective treatments, and designing vaccines.
2. *Interdisciplinary nature:* Virus taxonomy requires a multidisciplinary approach that involves experts from different fields, such as virology, systematic biology, evolutionary bioinformatics, genomics, structural biology and taxonomy, which can be challenging to coordinate. This is best illustrated by the confusion surrounding the naming of several emerging SARS-CoV-2 lineages. The World Health Organization (WHO), nextclade, PANGO, and even social media have referred to different variants with different labels. For example, SARS-CoV-2 variant BA.2.75 was initially nicknamed “Centaurus” on social media and the name was later picked up by both scientists and media.
3. *Nomenclature:* Nomenclature is the process of assigning unique identifiers (names) to viruses that would aid oral and written communication among scientists. ICTV administers nomenclature of ranks but not of names and abbreviations of viruses and their sub-classifications (e.g., isolates, strains, variants, lineages, clades), which fall within the purview of the International Code of Virus Classification and Nomenclature (ICVCN). Simmonds et al. (2023) effectively insists that naming viruses and virus taxonomic ranks should be unrestricted. Consequently, nomenclature used in virology can be confusing, error-prone and inconsistent. Once again, this is best illustrated with a SARS-CoV-2 example. The ‘official’ PANGO nomenclature uses an alpha-numeric system to name SARS-CoV-2 variants (e.g., B.1.1.529 for Omicron) and introduces new labels when the numerals go beyond three levels (e.g., BQ.1.1 is alias for B.1.1.529.5.3.1.1.1.1.1.1). As a result, variant evolutionary histories are not intuitively obvious from variant labels. Admittedly, classification below the species level remains an open question.
4. *Lack of culture systems and laboratory cross-validation:* Many viruses cannot be cultured in the laboratory, which makes it difficult to study their characteristics and classify them accurately. In particular, the decision by ICTV to accept metagenomic sequence data as sufficient evidence for the ‘discovery’, naming, and hence classification of viruses, has been turning point of concern in the field (Simmonds et al., 2017). Since then, a large majority of viruses “discovered” have not been isolated, and their existence is attested by partial genomic sequences assembled from increasingly large and complex sequence read datasets with constantly changing assembly programs. These programs use non-uniform sets of *ad hoc* parameters, none of which have been rigorously tested on controls of comparable complexity. The problem here, is multiple. The lack of physical/culturable isolates precludes the exchange of material between laboratories, once a set-in-stone requisite for microbiological validation. In most cases, the reproduction of the bioinformatic assembly/discovery process is not even possible, due to the huge computing resources required to process the large datasets (Gaïa et al., 2023). The term ‘discovery’, increasingly used in the context of metagenomic studies, is also unwarranted, as metagenomic viral-like sequences are only identified through their similarity with previously isolated viruses. Truly ‘new’ viruses remain undetected until a related prototype appears in the databases. These studies also tend to ignore the propensity of assembly programs to make many errors, making contigs from short identical nucleotide sequences, such as repeated sequences frequent in viral genomes, and creating large chimaeras leading to predicted unconfirmed record-sized genomes, for example for giant viruses (Schulz et al., 2017). Interestingly, the isolation of a virus belonging to this giant virus group by a different laboratory forced its classification according to the previous theoretical isolate (Klosneuvirus) and turned up to have a genome with much less impressive size (Deeg et al., 2018). The most extreme case of metagenomic-based taxonomic nomenclature is that of Mirusvirus, the chimeric nature of which was taken for granted (despite being a common error in large-scale sequence assembly) leading to the proposal of a new phylum dubbed *Mirusviricota*, which exhibits characteristics of two distinct realms, *Duplodnaviria* and *Varidnaviria* (Gaïa et al., 2023). ICTV is now compromising its own deepest ranking of dsDNA viruses based on what should be considered highly preliminary information. A quick fix for the distinction between theoretical versus isolated viruses would have been to retain the use of the prefix ‘candidate’ in front of all proposed names of uncultured viruses as it is norm for the classification of uncultured prokaryotes. Unfortunately, ICTV rejected the proposal for unknown reasons.
5. *Rapid evolution:* Viruses can evolve quickly, and new strains may emerge that are difficult to classify. Viruses, especially RNA viruses, are known for their high mutation rates, which can lead to rapid evolution and the emergence of new strains or subtypes. For example, SARS-CoV-2 mutation rates range 1-2×10^−6^ mutations per nucleotide per replication cycle, which is consistent with rates of other betacoronaviruses (Amicone et al., 2022). Rapid evolution makes it challenging to establish a stable and comprehensive classification system, as viruses can evolve and change quickly over time. For example, the A/H3N2 component of the Influenza vaccine has been updated 8 times between 2010 and 2022 and the SARS-CoV-2 vaccine will be updated for the 3^rd^ time in the 4^th^ year of pandemic (Kistler and Bedford, 2023). HIV-1 can generate enormous sequence diversity inside a single host even greater than the sequence diversity in humans in 2.5 million years of evolution (Leitner, 2018). Likewise, HIV-1 evolutionary rates differ among subtypes (Nasir et al., 2021b). Moreover, intra-host evolution and chronic infections can further accelerate the rates of evolution.
6. *Sequence divergence, hybridization, and lack of complete genome sequences:* Some viruses have highly divergent sequences, which can make it difficult to compare them to other viruses and classify them accurately. This is the case for the giant Pandoravirus, the prototype of which exhibited 93% of ORFans among its 2,556 protein-coding genes, and less than half of the genes consistently present in large dsDNA viruses (i.e., ‘core’ genes) (Philippe et al., 2013). In addition, viruses can undergo for example genetic recombination, pseudo-recombination, and hybridization, typical for example in the begomoviruses, a family of highly successful plant viruses (Chakraborty and Kumar, 2021; Fiallo-Olivé and Navas-Castillo, 2023). Such genomic divergence can further complicate virus classification. One example is the emergence of the SARS-CoV-2 XBB variant via recombination of two BA.2 sub-lineages that is now the dominant variant worldwide leading to WHO recommending vaccine manufacturers include a XBB component into their Fall 2023 vaccines (World Health Organization, 2023). Although advances in sequencing technology have made it easier to sequence viral genomes, there are still a majority viruses for which complete genome sequences are not available. This can make it challenging to compare and classify viruses, as important information about their genetic material may be missing or remains chimerically assembled.
7. *Diversity:* The number of known viruses is increasing rapidly, and there may be many more undiscovered viruses, which adds to the challenge of classifying them. In addition, newly discovered viruses sometimes extend the host range of their virus families. Such is the case of viruses in the *Asfarviridae* family, which were originally known to infect only mammals (e.g., causing swine fever) but that are now also infecting marine gastropod mollusks (abalone, *Haliotis discus discus*) (Matsuyama et al., 2020). One of the primary challenges in developing a universal virus taxonomy is the high degree of genetic diversity among viruses. The rapid mutation rates of many viruses can result in significant genetic divergence over relatively short periods. Additionally, the lack of a universal marker gene or set of genes for viruses makes it difficult to develop a consistent taxonomy based on genetic sequence data alone.
8. *Complex physiology and genetics:* Viruses are complex and diverse, which can make identification and classification more challenging. One example is the wide morphological, physiological and genetic diversity of archaeoviruses that live in extreme geothermal and hypersaline environments, including unique virion morphology, mechanisms of replication, maturation and virus release, and distinct genomic makeup (Dellas et al., 2014). Their proteins have limited sequence homology to that of other viral groups but their similarities can be disentangled with networks of gene families shared by different genomes (Krupovic et al., 2018).
9. *Host range:* Viruses often have a narrow host range, meaning that they can only infect specific organisms or cell types. This can make it difficult to compare viruses across different hosts, as their characteristics and behavior may differ significantly. Conversely, many viruses can infect a wide range of hosts, including bats, mammals, and mosquitoes (e.g., Rift valley fever virus), making it difficult to classify them based on their host specificity. However, large host jumps such as from Bacteria to Eukaryotes have never been observed, though bacterioviruses can infect the bacterial microbiome of eukaryotes, further complicating the relationships among organisms.
10. *Incomplete understanding of virus biology:* There is still much to learn about the biology of viruses. This includes their modes of transmission (e.g., the controversy surrounding whether SARS-CoV-2 is airborne or not), replication strategies, interactions with host cells, and seasonal behavior. For example, a genetic link to seasonal behavior of a winter virus has been recently identified in a longitudinal analysis of 12 million SARS-CoV-2 genomes (Tomaszewski et al., 2023). Viruses appear to tailor their genetic makeup according to latitude and temperature variations worldwide, suggesting a planetary integration of evolutionary trajectories. Without a complete understanding of virus biology, virus classification remains difficult and controversial, despite statements of virus taxonomists (Simmonds et al., 2023)

## Fallacies of virus taxonomy

Revisiting the three major battleground challenges of Figure 1 reveals that the initial problems we raised in the introduction for taxonomy in general are much more severe and complex for viruses. These problems often embody fallacies of different types, including argumentative, logical and cognitive (*appeal to probability, appeal to authority*), argumentative and cognitive (*Black Swan effect*, *association fallacy*), argumentative (*ad nauseam, cherry picking, begging the question*), and logical (*reification*, *post hoc ergo propter hoc*, *affirming the consequent*), some of which we highlight below. Understanding the permeability of scientific inquiry to values, such as beliefs, prejudices, preferences, and convictions, allows dissection of interferences with scientific objectivity within the context of discovery (the need to formulate theories) but more importantly within the context of justification (the need to determine their truth or falsity) (Alves, 2020). Such knowledge helps ensure research and scientific evidence will not become servant to ‘opportunistic interests’ or ‘authoritative or dogmatic attitudes’ (Alves, 2020). While a number of lessons can be drawn from the social sciences, more emphasis on epistemology can help acknowledge both the positive and negative influences that value interference has on the scientific endeavor.

### Holobiont-integrated viruses cannot be taxonomic units

Viruses are crucial contributors to the genomic and functional diversity of holobionts (Rosenberg and Zilber-Rosenberg, 2013; Grasis, 2017). Endogenous viruses transmit information vertically from one generation to the next, while virus infections transmit and rearrange information horizontally in holobiont collectives. Because viruses enter into obligatory intracellular interactions with their hosts, a significant fraction of cellular lineages are affected by their presence during the course of evolution. This reality was already advanced by Bandea (1983): *“viruses should be considered as organisms which develop their morphologically dispersed, physiologically active vegetative phase intracellularly, and which reproduce through inert unitary morphological forms, the virions.”* In fact, retroviral integrations have reshaped hologenomes. To illustrate, the human genome contains retrovirus fragments that make up over 8% of our DNA (Barton et al., 2009). While most of this viral DNA contains no discernable functions, some viral-encoded proteins have been fundamental. For example, Syncytin is required for the development of the placental syncytium and its evolutionary acquisition may have led to the formation of placental mammals (Dupressoir et al., 2009). Viruses might have also participated in the creation of eukaryotes, a superb example of entanglement in evolution: the parasite creating its own host! (Claverie, 2006; Claverie and Abergel, 2010). In viral eukaryogenesis, the cell nucleus of eukaryotes evolves from an endosymbiosis of a DNA virus with either a methanogenic archaeon or a bacterium (Villarreal and DeFilippis, 2000; Bell, 2001; Takemura, 2001). There is growing evidence supporting viral eukaryogenesis (Bell, 2020). For example, the assembly of a nucleus-like structure resembling a virus factory during bacteriophage 201φ2-1 replication in bacteria separate the viral DNA and proteins needed for DNA replication and transcription from the cytoplasm (Chaikeeratisak et al., 2017). The process involved a bipolar tubulin-like spindle, suggesting an ancestral viral link to nucleus formation. Consequently, virus evolution and classification cannot be disentangled from that of their hosts.

Current ICTV taxonomy borrows the traditional Linnaean classification scheme by perpetuating the notion that viruses are nothing more than a group of microbes sharing a set of homologous components (e.g., hallmark or core genes). This justifies grouping them together with phylogenetic and classification methodologies. However, this is fallacious. While viruses share an obligatory intracellular parasitic mode of life and a propagation/replication system that transitions through an apparently ‘inert’ macromolecular structure (the virion), the word ‘virus’ in its generality characterizes ‘a process’ and not something philosophically concrete. Using the word virus in the usual virological sense is a philosophical error called “reification,” the fallacy of treating an abstraction as if it were a real ‘concrete’ thing. In this context, any attempt of classification or phylogeny, loses its deep meaning and becomes absurd, like trying to classify religions from the objects manipulated during their cults. We note that Lwoff initially denied the notion that viruses possess a “living” nature in his historical papers. He based this famous denial on the absence of what he referred to as “organismal continuity” or the eclipse phase. However, this perspective arose from his confusion between the terms “virus” and “virion,” a confusion that persists among many virologists today. However, he also aligned himself with a processual view of viruses (Burnet, 1957): “*‘a virus is not an individual organism in the ordinary sense of the term, but something which could almost be called a stream of biological patterns’. I should like to say that I am in complete agreement with this statement which, by the way, is due to Sir MacFarlane Burnet”* (Lwoff, 1957). This view is taking hold (Claverie and Abergel, 2016; Dupré and Guttinger, 2016; Nasir et al., 2020). Therefore, viewing a virus as a concept rather than a tangible entity becomes essential.

While treating viruses as processes is aligned with ‘processual’ ontological views of biology (Bapteste and Dupré, 2013), it introduces difficulties and is therefore neglected in virus taxonomy. If a classification at a given rank brings together entities (e.g., organisms) with common basic functionalities (often a mode of reproduction), classification of an entity at a given level must allow functional predictions on other entities classified at the same level. For viruses, the intracellular replication mode is one of these basic functionalities. For example, *Bamfordviridae* includes viruses with purely cytoplasmic, nuclear, or mixed replication. Similarly, the presence/absence of a transcription system becomes an extremely strong classification criterion. For example, viruses can code and load (e.g., Mimivirus), code but not load (e.g., Marseillevirus), or not encode a RNA polymerase (Chlorella viruses). Such gradation supports monophyly if one adopts the genomic reduction scenario of progressive loss of function that is currently rejected by nomenclators. Another example is the asymmetry between the presence or absence of DNA polymerase and RNA polymerase. No DNA virus has been identified with an RNA polymerase but no DNA polymerase. Replication must pass through the host nucleus and the asymmetry explained by a progressive loss of function dictated by a yet-to-be determined evolutionary process.

Thus, viruses cannot be considered *bona fide* taxonomic units while at the same time their convoluted evolution cannot be ignored by phylogeneticists and taxonomists alike. This challenges the entire taxonomic endeavor.

### Primacy of paraphyly (not monophyly-polyphyly) in phylogeny and virus evolution

There are significant disagreements about the centrality of monophyly and the rejection of paraphyly in biological classification (Podani, 2010). *Monophyly* is the taxonomic grouping of a common ancestor and all of its descendants on a phylogenetic tree (or a taxon in classification). This *monophyletic* relation (also known as ‘clade’) contrasts with *paraphyly*, a grouping that contains the common ancestor but excludes some of its descendants. Many taxonomists and pattern cladists consider monophyly is the only valid grouping for classification (e.g., Simmonds et al., 2023), while others (including evolutionary taxonomists and process cladists) think paraphyly is desirable, tolerable, unavoidable or unacceptable (Podani, 2010). If classification adopts evolutionary principles, two approaches can be taken: (i) divide a tree into clades, nesting them with each other (the approach of *PhyloCode*; de Queiroz and Cantino, 2020) but then disregard reliance on taxonomic ranks such as families and genera; or (ii) use phylogenetic characters to distinguish mutually exclusive and ranked taxa (the approach of evolutionary classification), which requires acceptance of paraphyletic relationships and rank-based codes (Brummit, 2008). Regardless of debate or stance, many paraphyletic relations exist in reconstructed phylogenetic trees that seek explanation. Some are of crucial significance. For example, when building a ToL, modern phylogenetic analysis favors reconstruction of unrooted trees because (i) the space of possible unrooted trees is smaller and computationally more tractable than the space of rooted trees, (ii) there is no outgroup available that can be used to root the monophyletic ToL construct, (iii) optimization-based polarization with ultrametric distances that exhibit ‘molecular clock’ properties often fail the triangle inequality condition that impacts the validity of phylogenetic reconstruction; and (iv) midpoint rooting and parametric-based rooting methods are either highly sensitive to unbalanced rate heterogeneities, biased, or dependent on ultrametricity in data, an absence of which compromises parametric maximum likelihood or Bayesian methodologies (Caetano-Anollés et al., 2018). Yet, the powerful ‘generality criterion’ embodied in Weston’s rule, when used *a posteriori*, can offset most problems listed above. For example, given an unrooted ToL showing all three organismal domains as monophyletic, pulling down a branch most parsimoniously with the Lundberg optimization method defaults into basal paraphyletic relationships when the branch is part of a putative monophyletic group. That is exactly the case of a ToL reconstructed from a survey of structural domains in entire proteome complements (e.g., Wang et al., 2007) that is rooted with Weston’s rule. In such reconstructions, domain Archaea is placed at the base of the ToL as a paraphyletic group [reviewed in Staley and Caetano-Anollés (2018)]. A similar paraphyletic placement is obtained when a rooted ToL is reconstructed from Gene Ontology (GO) definitions of molecular function (Kim et al., 2014). Since these phylogenetic reconstructions of rooted trees with powerful optimality criteria are robust and congruent, the resulting paraphyletic groupings must be appropriately interpreted to gain further evolutionary insight. We contend the initial grades that appear as off-shoots at the base of the ToL likely represent the products of a process of gradual reductive evolution leading to the highly reduced proteome repertoires of modern Archaea. These processes are the likely result of information compression (Caetano-Anollés, 2021). They could also represent primordial evolutionary grades (*sensu* Huxley, 1958), i.e., groups of diversifying organisms in active transition that were initially unified by similar physiological complexities of primordial archaeons that were emerging from the ancestral stem. The existence of basal paraphyletic groups may also result from multiple origins established at the beginning of primordial lineages. As suggested by Woese (1998, 2002), a communal cellular world fostering multiple origins likely arose prior to or during the time of LUCA from massive episodes of horizontal exchange. Unremarkably, the reconstructed ToLs show monophyletic groups of archaeons arising as clades from the basal paraphyletic groupings. Thus, monophyly and paraphyly coexist, are not mutually exclusive, and are emergent. In fact, they are plainly evident when diachronous classifications are overlapped onto phylogenetic trees according to Figure 1 in Podani (2010). Their joint presence cannot be disentangled, changing instead the definition of taxa and therefore complicating taxonomic classification.

**Figure 5:**
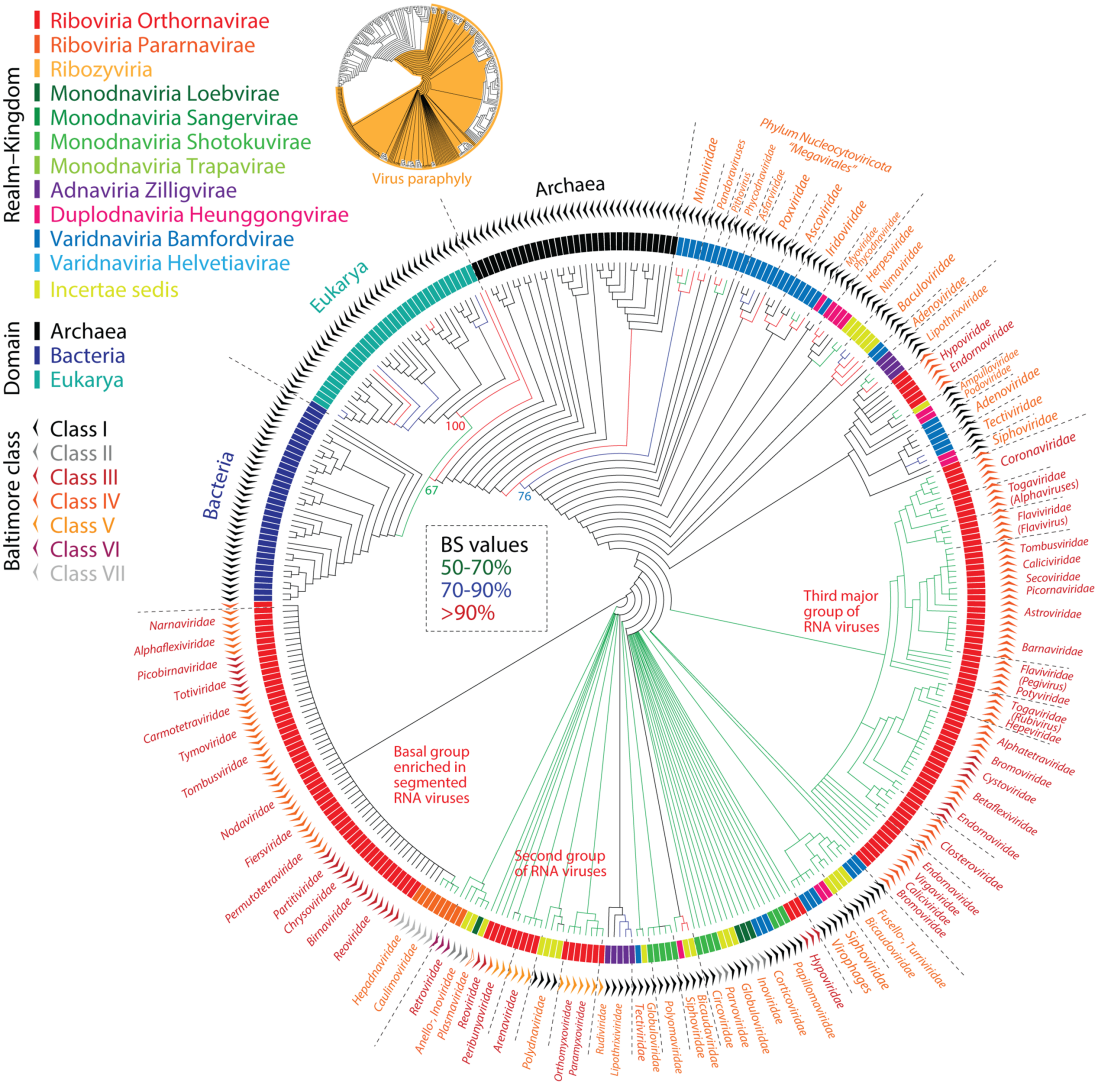
The basal paraphyletic grouping of viruses in a uToL describing the evolution of proteomes from and cellular organisms challenges the monophyletic classification of viruses. The phylogeny (phylogenetic tree length = 45,935; retention index = 0.83; g1 = −0.31) rendered in ‘fan’ format describes the evolution of 368 proteomes (taxa) randomly sampled from cells and viruses (Nasir and Caetano-Anollés, 2015). This tree of proteomes was reconstructed from 442 parsimony-informative phylogenetic characters representing genomic abundance of 442 domain structures that were universally present in the 3 domains of cellular life and viruses and were defined at SCOP fold superfamily level of protein classification. Differently colored branches represent bootstrap support (BS) values. Viral taxa are labeled with family names and are indexed with realms-kingdoms and Baltimore classes. While many viral families do form largely unified monophyletic groups, viruses as a collective group is paraphyletic and so are most realms or Baltimore classes. Insert: Virus paraphyly in deep branches leading to virus families are traced in orange.

Paraphyletic relationships are also evident in a universal ToL that includes viruses (Nasir and Caetano-Anollés, 2015). In this uToL, viruses appear at the base of the rooted tree as a paraphyletic group followed by paraphyletic Archaea and then by monophyletic Bacteria and Eukarya (Figure 5). The same evolutionary processes that explain paraphyletic relationships in Archaea can be invoked for viruses, including reductive evolution, horizontal exchange, and recruitment. The primacy of the virus reductive mode of evolution is particularly significant. Tell-tale signs of reductive evolution include the fact that members of the entire virus supergroup enter into obligatory relationships with their hosts, that a wide diversity of viruses have patchy and highly reduced genomic repertoires, and that the genomes of giant viruses resemble those of bacteria with parasitic lifestyles. Tracing realms and kingdoms of viruses as well as Baltimore classes onto the uToL provides interesting insights about paraphyly and monophyly in virus evolution (Figure 5). Tracings realms onto terminal branches show that they do not make monophyletic groups. Instead, their appearance is spread in groups throughout the paraphyletic basal ensemble. *Riboviria* is split in at least 5 groups (some paraphyletic), *Monodnaviria* in at least 5, *Adnaviria* in 2, *Duplodnaviria* in at least 5, and *Varidnaviria* in 8. The basal placement of *Riboviria* in the rooted uToL tree is congruent with its basal placement in the tree of realms of Figure 3C. While tracing the more granular kingdoms fails to increase monophyly in the tree, tracing Baltimore classes also showed their paraphyletic disposition. Overall, the tracing exercise indicates taxonomies of realms, kingdoms and Baltimore classes do not reflect virus proteome evolution.

The validity of deep taxonomic ranks has been also challenged at more granular level. The phylogenomic analysis of plankton-infecting DNA mirusviruses of the phylum *Mirusviricota* has recently questioned the monophyly of *Realms* (Gaïa et al., 2023). The genomic repertoire of mirusviruses was found to be complex and chimeric, holding a genomic module of virion morphogenesis typical of herpesviruses of the realm *Duplodnaviria* and an informational module closely related to large and giant viruses of the realm *Varidnaviria*. The mirusvirus chimeric makeup suggests episodes of massive horizontal transfer between lineages but also a deep and planktonic ancestry of eukaryotic duplodnaviruses. Remarkably, this deep but close ancestry is reflected in the relatively close placement of herpesviruses and giant viruses in the uToL of Figure 5. Since a phylum of a virus cannot belong to two realms at the same time, each of which are assumed to be monophyletic and with separate origins, the *Realm* classification as it now stands must be revised. Monophyly has also been challenged at the *Kingdom* level with double stranded DNA virus of the realm *Varidnaviria* (Woo et al., 2021). A sequence-based phylogeny of concatenated major capsid proteins and packaging ATPases revealed that *Sphaeolipoviridae*, the only virus family of kingdom *Helvetiavirae,* had a chimeric origin, with capsid proteins grouping with kingdom *Helvetiavirae* and packaging ATPases grouping with those of kingdom *Bamfordvirae*. A similar exploration, this time focused on the double-jelly roll capsid structure, supports a separate origin of the two kingdoms of *Varidnaviria* (Krupovic et al., 2022) and the conclusion: *“Thus, revision of the realm Varidnaviria seems to be due. The continuing accumulation of sequence and especially structural data on cellular and viral proteins is bound to entail further refinement of the scenarios of the origin and evolution of each of the major groups of viruses, and the corresponding changes in virus taxonomy*.”

The current ICTV-vetted ‘megataxonomy’ of viruses considers most *Realms* are polyphyletic (Koonin et al., 2020). *Polyphyly* is an atypical grouping where members do not share an immediate ancestor (Podani, 2010). The grouping is rejected for classification by taxonomists in overwhelming consensus. The standard definition of a polyphyletic relation is a group that does not include a common ancestor and all of its descendants, usually in the form of organisms occurring on different branches of a tree and having different most recent ancestors. Obviously, all organisms are unified by a classical ToL, so polyphyly is a relative concept and ultimately resolves as sets of monophyletic and paraphyletic relations. Polyphyly in viruses however has been given a different evolutionary undertone. Since alignment-dependent phylogenetic methodologies are unable to unify the virus world, single or sets of hallmark genes are used to build monophyletic groups that lack common ancestors. These highly ranked polyphyletic entities are not explained by the methodological limitation of using sequence-based phylogenetic methods to dissect a highly patchy virus world. Instead, the groups are rationalized as originating in separate manner from different ancestral replicators (Koonin et al., 2021). This view is clearly incompatible with structural phylogenomic data used to reconstruct the uToL of Figure 5, which supports the existence of LUCA and other ancestors of modern life.

Claiming that shared homologies are the result of vertical evolution can be questionable, especially in light of the reticulated and highly dynamic evolutionary changes that are typical of viruses. In fact, dissecting evolutionarily deep phenomena rests on proper corroboration of homology definitions (Ochoterena et al., 2019) and proper use of retrodiction methodologies (Caetano-Anollés et al., 2018; Caetano-Anollés, 2023). In this regard, the application of alignment-dependent phylogenetic methods to explore the evolution of a limited set of virus hallmark genes must be conducted and interpreted with extreme caution. For example, Wolf et al. (2018) unified the highly divergent groups of RNA viruses with a phylogeny reconstructed from aligned sequences of the highly conserved RdRp polymerase enzyme. The study resulted in a proposal for *Riboviria*, contending support for a ‘virus-first’ model of viral origins and an ancient monophyletic group of viruses (Koonin et al., 2020). However, a re-evaluation of their alignment, encompassing 4,627 taxa and 12,220 amino acid sites, questioned its ability to accurately capture RNA virus evolution (Holmes and Duchêne, 2019). Problems with the alignment included the existence of a gap in every aligned site, absence of contiguous aligned stretches across all taxa, only 3.6% of the alignment (441 amino acid residues) kept after trimming sites with >50% gaps, pairwise identity between aligned sequences being less than the 5% expected by chance, 812 sites containing all 20 amino acids, 95.9% of sequences failing a test of compositional heterogeneity, and finally, only 6 or no sites being validated as being alignment-safe by two trimming-validation programs using the most permissive settings. This illustrates the perils of pushing the evolutionary limits of alignment-dependent reconstruction methods. The tree of Wolf et al. (2018) was rooted using reverse transcriptases from Group II introns and non-LTR retrotransposons as outgroups, which assumes their ancestral relatedness to RdRp, or with the midpoint rooting procedure, which as previously mentioned is highly susceptible to deviations from a constant evolutionary rate, especially in an unbalanced tree like the RdRp phylogeny. Since viruses of other realms could not be included in the analysis (they lack the enzyme), the tree cannot be used to support the monophyly of *Riboviria*, questioning the rationale for the existence of such a Realm. The RdRp phylogeny established 5 ‘branches’, 2 harboring only Baltimore class IV viruses (branches 1 and 3), one with a mix of class III and IV viruses (branch 2), another harboring class III viruses (polyphyletic branch 4), and a final group with class V viruses (branch 5). By far, the families of *Riboviria* are the most popular in the uTol of Figure 5. They make at least 3 major groups, a basal group that is enriched in segmented RNA viruses with class III and IV replication strategies, a second group of class III and V viruses appearing together with class I and II DNA viruses, and a third major group enriched in class IV RNA viruses that makes up a paraphyletic ensemble of several monophyletic family groups. The groupings in the RdRp and uToL are not congruent, suggesting sequence and structure carry different phylogenetic signatures.

### Structural phylogenomic analysis challenges the polyphyletic scenario of multiple viral origins

**Figure 6:**
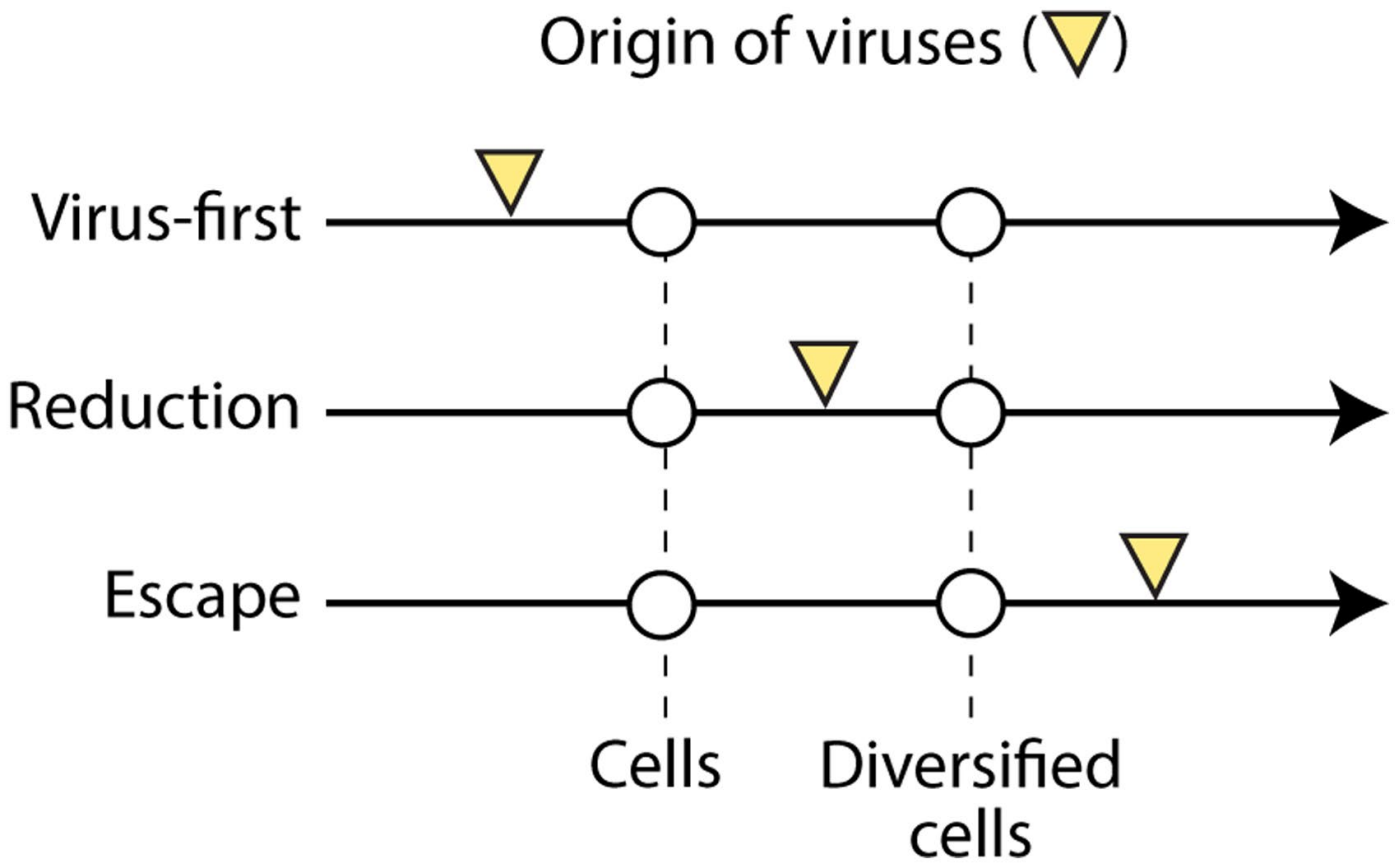
Three main scenarios of viral origins suggest viruses originated during either a pre-cellular world, a primordial cellular world, or a diversified cellular world. The pre-cellular ‘Virus-first’ hypothesis is problematic because all viruses depend on cells to propagate. The ‘Escape’ hypothesis in which viruses originate as ‘escapees’ from already diversified cells belonging to Archaea, Bacteria or Eukarya, is incompatible with viruses carrying conserved protein fold structures that are common to all domains of life, which suggest they arose prior to the ‘last universal cellular ancestor’ (LUCellA). The more likely ‘Reduction’ hypothesis suggest viruses appeared prior to LUCellA in an emergent cellular world.

The origin and early evolution of viruses impacts the validity of deep taxonomic ranks but remains an unsettled problem in biology. While three general scenarios of origin have been proposed over recent years [Figure 6; reviewed in Nasir et al. (2012a, 2020)], most hypotheses associated with these frameworks lack explanatory power, only few if any have been debated, and more recently, some have been heralded *ad nauseam* without considering mounting countering evidence (Krupovic et al., 2019). In the ‘virus-first’ scenario of viruses being ancestral to cells (D’Herelle, 1922), viruses originate from prebiotic pools of replicators during a pre-cellular world (Koonin et al., 2006; Koonin and Dolja, 2014). This framework assumes that nucleic acid replicators appeared in absence of cellular makeup, proteins, or translation machinery. This is in itself problematic because the ancient RNA world that supports these ideas has been seriously contested on many grounds (Kurland, 2010; Bernhardt, 2012; Caetano-Anollés and Seufferheld, 2013). In addition, the tight dependence of virus propagation on protein replication and cellular machinery makes it difficult to envision how nucleic acid-based replicators (ribozymes) could have integrated their replication abilities into protein and cellular makeup. In the ‘reductive’ evolutionary scenario, viruses originate from primordial cells fully integrated into an emergent cellular world (Bandea, 1983, 2009; Claverie, 2006). With time, their cellular makeup becomes compressed by processes of reductive evolution in ways resembling those typical of obligate parasites. Early hypotheses supporting the ‘reductive’ scenario exist that differentiate between cell-like and parasite-like stages of virus evolution [e.g., the ‘extrusion’ model of Nasir and Caetano-Anollés, (2021)] or pathways to replication (Nasir et al., 2020). The discovery of giant viruses with genomic and structural features typical of cells [reviewed in Colson et al. (2017)] and data-driven structural phylogenomic analyses (Nasir et al., 2012b, 2015; Nasir and Caetano-Anollés, 2015; Colson et al., 2018; Mughal et al., 2020) support these types of hypotheses. In the ‘escape’ scenario, viruses originate from rogue genetic entities that escaped cellular control in a modern diversifying cellular world (Moreira and Brochier-Armanet, 2008; Moreira and López-García, 2009). These molecular escapees later evolved by borrowing useful cellular genes via horizontal gene transfer processes. The model, which is supported by homologies between a small set of virus and host genes, explains why viruses have hosts that are specific to them and exchange genes preponderantly with hosts of their own cellular domains (Malik et al., 2017). However, the escape scenario cannot explain genes unique to viruses, genes poorly represented in cells, viral genes that are present in all domains of life, or genes that resist annotations. Hybrid models that combine pre-biotic replicators of the ‘virus-first’ type and ‘escape’ events explaining protein folds of viral capsids (Krupovic et al., 2019), are now being used to propose deep taxonomic ranks (Koonin et al., 2020, 2021).

**Figure 7:**
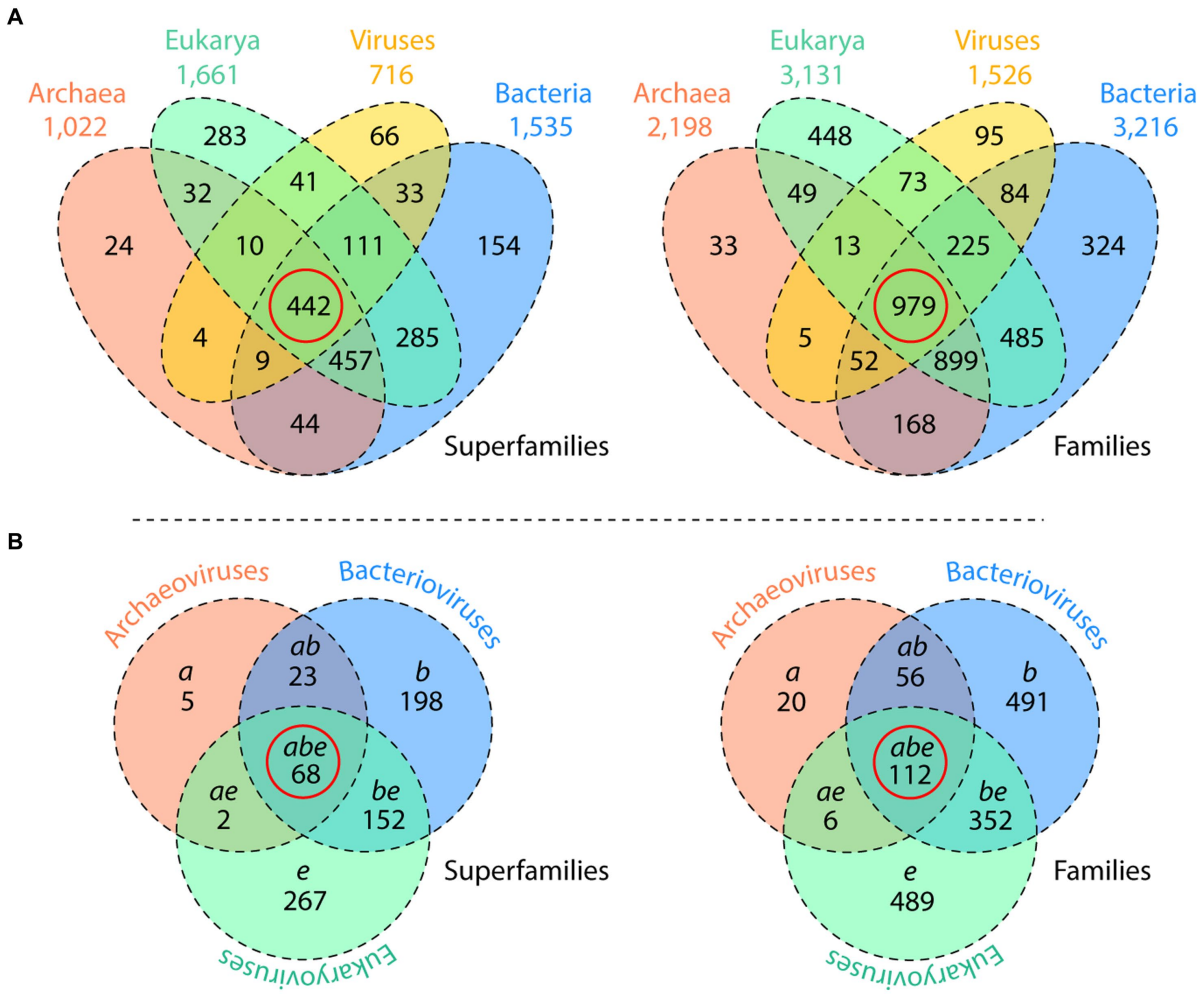
A census of SCOP structural domains challenges the ‘virus-first’ and ‘escape’ hypotheses. **(A)** Venn diagrams describe the distribution of 1,995 fold superfamilies and 3,892 fold families identified with HMMs of structural recognition in Archaea, Bacteria, Eukarya, and viruses following a survey of 5,080 and 8,127 proteomes, respectively. The red circle highlights the number of superfamilies and families that are shared by all three organismal domains and viruses. **(B)** Venn diagrams describe the distribution of the 715 superfamilies and 1,526 families that were present in archaeoviruses, bacterioviruses and eukaryoviruses. Note that the existence of structures present in the three viral groups (the *abe* Venn group in the red circle) does not imply they belong to viruses capable of infecting organisms in the three domains of cellular life (an impossibility). Instead, it shows the groups of structural domains shared by viruses infecting the different hosts. Data from Nasir and Caetano-Anollés (2015) and Mughal et al. (2020).

Support for hypotheses of viral origins is expected to be drawn from the extant molecular makeup of viruses and their hosts, phylogenomic reconstruction, and inferences derived from chronologies of molecular repertoires, all of which must derive congruent predictions. In general, the ‘virus-first’ and ‘escape’ scenarios draw support from alignment-dependent phylogenetic methodologies while the ‘reductive’ scenario mainly rests on alignment-free methods. However, standard alignment-dependent methods are not suited for deep phylogenomic explorations because the genomic and proteomic makeup of viruses is patchy and hallmark genes cannot dissect virus origins. A focus on the more conserved structure of proteins and nucleic acids (Caetano-Anollés and Nasir, 2012) and the use of molecular structure in phylogenetic analysis with alignment-free methodologies promises better insight into deep evolutionary phenomena. We first illustrate this fact with a simple census of structural domains in proteomes, which already challenges the ‘virus-first’ and ‘escape’ hypotheses. Figure 7A shows Venn diagrams describing the distribution of 1,995 known structural domains defined at fold superfamily level of SCOP classification in 5,080 proteomes from 122 archaeal, 1,115 bacterial, and 383 eukaryal organisms and 3,460 viruses (Nasir and Caetano-Anollés, 2015) and the distributions of 3,892 structural domains defined at the more structurally conserved fold family level in 8,127 proteomes from 139 archaeal, 1,734 bacterial, and 210 eukaryal organisms and 6,044 viruses (Mughal et al., 2020). These SCOP superfamilies and families approximate the diversity of the world of proteins, as very few folds are expected to be newly discovered. Remarkably, the largest Venn groups of fold structures were shared by Archaea, Bacteria, Eukarya and viruses (the ABEV group) or by Archaea, Bacteria and Eukarya (ABE). In absence of horizontal transfer of genetic information, these results support the existence of both a common ancestor of viruses and cells and a common ancestor of cells, especially because the spread of individual fold structures in cells and viruses was found to be substantial. Besides the significant numbers of common ABEV and ABE structures, the viral supergroup encompassed 715 superfamilies and 1,526 families with Venn distributions comparable to those of cellular domains, highlighting the structural complexity that exists in viruses and providing further support to a cellular origin of viruses. More remarkable is the large number of virus-specific fold structures (66 superfamilies and 95 families), which were larger in number than Archaea-specific counterparts. Within the 715 superfamilies and 1,526 families of the viral supergroup, there was a significant core set of fold structures that was shared by viruses infecting Archaea, Bacteria, and Eukarya (Figure 7B). The existence of cores of 68 superfamilies and 112 families shared by archaeoviruses, bacterioviruses and eukaryoviruses (the *abe* groups) supports the existence of a common ancestor to all viral groups. These structures were detected in a large number of viruses from each Baltimore replicon type and were responsible for crucial metabolic functions. They were widely shared by organisms in all domains of cellular life, judged by a significant spread of fold structures in the proteomes of Archaea, Bacteria and Eukarya (measured with an *f*-index that describes the fraction of taxa holding a phylogenetic character).

**Figure 8:**
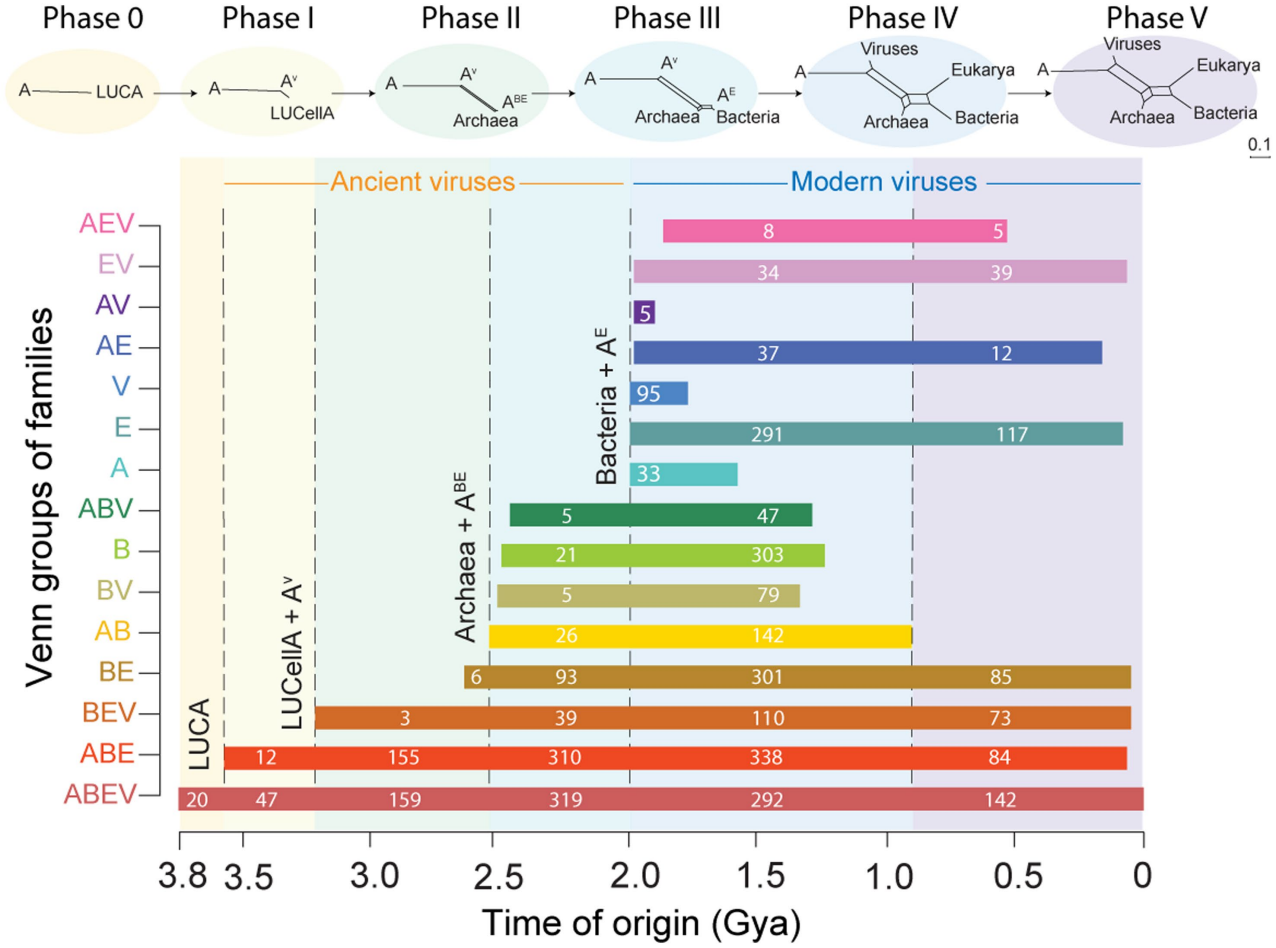
The evolutionary history of structural domains defined at SCOP family level reveals gradual evolutionary accumulation of domains in the proteomes of cells and viruses. A rooted phylogenomic tree describing the evolution of the 3,892 families that are present in 8,127 proteomes allowed calculation of times of origin for families unique or shared among Archaea (A), Bacteria (B) and Eukarya (E) and viruses (V). Horizontal bar plots show ranges of ‘times of origin’ in a geological time scale defined by a molecular clock of folds that ranges from the origin of domains 3.8 billion years ago (Gya) to the present (0 Gya). Numbers in bars indicate families appearing in each evolutionary phase of the timeline. A most likely chronology of cellular evolution inferred from Venn group distributions is shown on top of bar plots as a series of phylogenetic networks reconstructed with the Neighbor-Net algorithm in SplitsTree. The chronology confirms an evolutionary progression in which ancestral cells (A) coalesce into a last universal common ancestor (LUCA), which then diversifies into a last universal cellular ancestor (LUCellA) and ancestors of viruses (A^V^), the rise of Archaea and a stem line leading to ancestors of Bacteria and Eukarya (A^BE^) and then Eukarya (A^E^), and finally to modern diversified lineages of Archaea, Bacteria, Eukarya and viruses. A similar progression was obtained when analyzing domains defined at superfamily level. Data from Mughal et al. (2020).

While comparative analyses of census data falsify the heralded polyphyletic scenario of few or a multitude of independent viral origins (Koonin et al., 2023), the phylogenomic reconstruction of rooted trees of structural domains with alignment-free methods confirmed these inferences and provided further evidence supporting the cellular origin of viruses (Nasir et al., 2012b, 2015; Nasir and Caetano-Anollés, 2015; Colson et al., 2018; Mughal et al., 2020). Chronologies describing the origin and evolutionary accumulation of structural domains in proteomes derived from trees of domains rooted with the generality criterion and Lundberg revealed strong vertical evolutionary signatures [reviewed in Caetano-Anollés et al. (2021)]. We illustrate their power with a chronology describing times of origin of SCOP families unique or shared among domains of life and viruses (Figure 8). Six evolutionary phases unfolded along the evolutionary timeline. As expected for a system diversifying by vertical descent, the most ancient phase (Phase 0) was found to hold domain structures belonging to the universal ABEV Venn group common to cells and viruses. These domains make up proteins linked to membranes and genetic code specificities encoded in a ‘pangenome’ of an ancient communal cellular world. Expectedly, the second oldest phase (Phase I) harbored younger ABEV and ABE structures (many typical of ribosomal and cell adhesion proteins) that signaled the rise to two stem lines of descent from a last universal common ancestor (LUCA), one leading to a last universal cellular ancestor (LUCellA) and another driven by reductive evolution leading to ancient cell-like viruses. In the third oldest phase (Phase II), the ABEV, ABE, BEV and BE domain repertoires indicate LUCellA diversifies by reductive evolution (and membrane phospholipid makeup) into ancestors of Archaea and a stem line common to Bacteria and Eukarya. In Phase III, the first structures specific to a domain of life (Bacteria) make their appearance and in Phase IV structures specific to the other domains of life and viruses become evident in the phylogeny, including the appearance of 95 virus-specific families harboring capsid and coat folds necessary for viral infection. Results therefore suggests parasitism appeared quite late in virus evolution. The chronology confirms an evolutionary progression that is only compatible with the reductive scenario of viral origins. It also falsifies the existence of an ever-increasing multiplicity of viral origins (Koonin et al., 2023), countering the promise to multiply the number of Realms in the virus classification: *“We argue that viruses emerged on a number (even if far from astronomical) independent occasions, so the number of realms will considerably increase from the current 6, by splitting some of the current realms, giving the realm status to some of the currently unclassified groups”* (Koonin et al., 2023).

**Figure 9:**
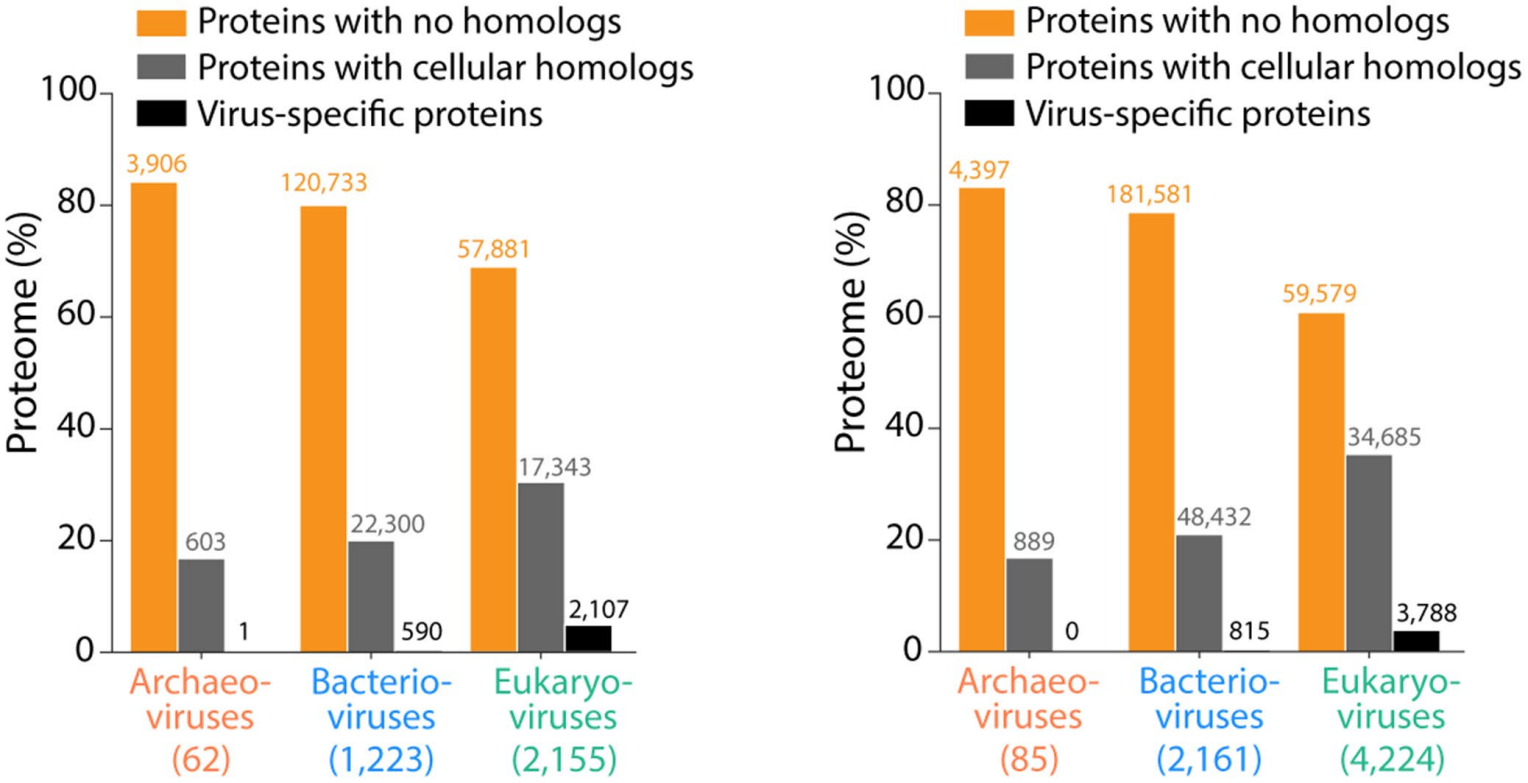
Proteomic composition of viruses infecting the three domains of cellular life. Numbers in parentheses indicate number of virus that were surveyed. Data from Nasir and Caetano-Anollés (2015) and Mughal et al. (2020).

Finally, one remarkable finding of comparative genomic analysis of viruses is that most proteins lack detectable homologs and domain assignments (Figure 9). About 80% of proteins from prokaryotic viruses and about 65% of proteins from eukaryotic viruses represent ORFans, while the rest of the genes were either encoding proteins with cellular homologs or virus-specific proteins. All of these comparative genomic patterns provide a strong indication of an ancient origin of viruses in coevolutionary interaction with cells. Common cores are not compatible with views of multiple origins from a pre-cellular world (unless different primordial replicators converged toward a common ancestor during cellular emergence) or rogue elements capturing genes from modern cells. In fact, the genetic majority of ORFans making up the viral genome suggests the opposite, that viruses are actual donors of genetic novelties to cells, eventually through the *de novo* creation of genes (Legendre et al., 2019).

Thus, comparative genomics and structural phylogenomic analysis of thousands of proteomes from cellular organisms and viruses are incompatible with the proposal of selfish nucleic acid replicators recruiting cellular makeup in the form of capsid proteins to form modern viruses that support the deep ranks of current ICTV virus taxonomy. This conclusion is aligned with semantics. If words have meanings, a ‘virus’ is an obligate parasite of cells, *“a submicroscopic infectious agent that replicates only inside the living cells of an organism”* (Wikipedia definition). Then, how would viruses emerge before cells, or at least, before an ancestor of modern cells. Perpetuating Lwoff’s ‘virus’ and ‘virion’ confusion is at odds with the mounting view of viruses defining a cellular ‘process’, not a material object or a living entity. The process involves sharing a way of replication through the making of infectious inert particles. Thus, an ancient cellular (eventually multiple) origin of viruses seem the only way forward but challenges the validity of current deep ICTV taxonomic ranks.

## Conclusions and recommendations

Linnaean taxonomies organize species in a pyramidal taxonomic structure that follows a ‘subsumption’ (specification) hierarchy of nesting relationships of the ‘is-a-kind-of’ type (Salthe, 2012). This contrasts with the other logic form of hierarchy, the ‘compositional’ hierarchy of nesting relationships of the ‘is-a-part-of’ type typical of mereological descriptions of systems. The Linnaean subsumption hierarchy is based on genotype and phenotype features shared by taxa, with low-level Linnaean categories (e.g., genus, family) sharing more granular details of properties of taxa and higher-level categories (e.g., order, class) sharing fewer and more broader descriptions. Three major ontological, epistemological and methodological assumptions support the Linnaean subsumption hierarchy (Salthe, 2012). The main ontological assumption is that every taxonomic entity had to develop from earlier and simpler conditions as part of either a developmental or evolutionary trajectory. The main epistemological assumption is that in order to understand a taxonomy that represents a specific system (e.g., organisms, viruses) there is a need to look for its sources in prior systems. Finally, the main methodological assumption is that information about the system being classified can be found in ‘antecedent’ conditions (perhaps ancestral), which unfold as discrete stages or series of ancestral types. Thus, philosophical arguments demand that Linnaean taxonomies search for an increasingly historical rationale. We have seen how this demand is being adopted by modern taxonomy, which has embraced the use of cladistic approaches to organize species on an evolutionary basis driven by time and ‘shared and derived’ features describing descent with modification. There is consensus: “neglecting evolution is bad taxonomy” (Hörandl, 2007). It is clear that fulfilling the evolutionary demand for virus taxonomy has been one driver of ICTV (Simmonds et al., 2023). Given our critical appraisal, the onus is on ICTV to address concerns we have raised by taking more conservative paths to classification, such as reverting the taxonomic classification of viruses to a lower ranked system of the type that precedes the ICTV release 2018 (MSL #34), which is aligned with the first taxonomic proposals of Lwoff et al. (1962), and considering viruses as processes with functions that must be integrated with those of their hosts. The impact of viruses as holobiont agents must be carefully evaluated as well as the effect of horizontal exchange of genetic information, always adopting the most conservative strategy of taxonomic classification that shields against violations of evolutionary history and biological organization. Phylogenetic reconstruction must search for more conserved phylogenetic characters that capture the history of increasingly broader virus groups, acknowledging alignment-dependent methods that solely focus on sequence and the structure of individual folds will only dissect the shallow history of close relatives (at the family level). Finally, increasingly better computational methods of phylogenetic reconstruction must be sough that are capable of better dissecting episodes of evolutionary reticulation (and not implying/forcing tree-like structures).

## Author contributions

GC-A, J-MC, and AN contributed to the conception and design of this critical review. GC-A wrote the first draft of the manuscript. All authors contributed to manuscript revision, improvement, and approval of the submitted version.

## Funding

Research has been supported by grants from the National Institute of Food and Agriculture, the Illinois Campus Cluster Program (ICCP), and Blue Waters supercomputing allocations from the National Center for Supercomputing Applications (NCSA) to GC-A, and by recurrent funding from CNRS and Aix-Marseille University (IGS) to C. Abergel and the IGS laboratory.

## Conflict of interest

AN is a shareholder and employee at Moderna, Inc.

The remaining authors declare that the research was conducted in the absence of any commercial or financial relationships that could be construed as a potential conflict of interest.

## Publisher’s note

All claims expressed in this article are solely those of the authors and do not necessarily represent those of their affiliated organizations, or those of the publisher, the editors and the reviewers. Any product that may be evaluated in this article, or claim that may be made by its manufacturer, is not guaranteed or endorsed by the publisher.
